# Disseminated Herpes Zoster Following Protein Subunit and mRNA COVID-19 Vaccination in Immunocompetent Patients: Report of Two Cases and Literature Review

**DOI:** 10.3390/medicina59091542

**Published:** 2023-08-25

**Authors:** Chia-Shuen Lin, Chung-Hsing Chang

**Affiliations:** 1 Department of Dermatology, Hualien Tzu Chi Hospital, Buddhist Tzu Chi Medical Foundation, Hualien 97002, Taiwan; 101311127@gms.tcu.edu.tw; 2 Doctoral Degree Program in Translational Medicine, Tzu Chi University and Academia Sinica, College of Medicine, Tzu Chi University, Hualien 97004, Taiwan; 3 Institute of Medical Sciences, College of Medicine, Tzu Chi University, Hualien 97004, Taiwan

**Keywords:** disseminated herpes zoster, immunocompetent patients, mRNA, protein subunit, COVID-19 vaccination

## Abstract

Disseminated herpes zoster (DHZ), resulting from the reactivation of the varicella-zoster virus (VZV), typically occurs in immunocompromised persons. To date, only four cases of DHZ following mRNA, viral vector, or inactivated COVID-19 vaccinations have been reported in immunocompetent patients. Herein, we present the first case of DHZ following the protein subunit COVID-19 vaccination (case 1, 64 years old) and a case of DHZ following mRNA COVID-19 vaccination (case 2, 67 years old) in elderly, immunocompetent male patients. Both cases were generally healthy, without a remarkable underlying disease and without a history of immunosuppressant use. Case 1 developed DHZ (left C3–5 predominant) 1 month after receiving the third dose of the SARS-CoV-2 spike protein vaccine (MVC-COV1901). Case 2 developed DHZ (right V1–3 predominant) 7 days after receiving the second dose of the mRNA-1273 SARS-CoV-2 vaccine. Through skin examination, Tzanck smears, and dermoscopy, the diagnosis of COVID-19 vaccination-related DHZ was established in both cases. Oral famciclovir (250 mg, three times/day for 7 days) was administered, and both cases achieved total remission of skin lesions without visceral involvement or severe post-herpetic neuralgia. Our cases demonstrate that DHZ, as a rare cutaneous adverse event in immunocompetent patients, can be secondary not only to mRNA COVID-19 vaccination but also to the protein subunit COVID-19 vaccination. It is speculated that the spike protein of SARS-CoV-2 could be the common trigger for the reactivation of VZV among different types of vaccinations.

## 1. Introduction

With the widespread application of COVID-19 vaccines worldwide, diverse cutaneous adverse events have been reported [1,2]. Herpes zoster (HZ), resulting from the reactivation of the varicella-zoster virus (VZV), is a frequently reported cutaneous manifestation [1,2,3,4]. Disseminated HZ (DHZ), a rarely severe form of HZ, is defined by the presence of >20 vesicles beyond the primary or adjacent dermatome, which typically occurs in immunocompromised persons [5]. To date, only four cases of DHZ following COVID-19 vaccinations have been reported in immunocompetent patients [6,7,8,9] (Table 1). Among them, two cases were associated with mRNA-based vaccination [6,7], whereas the other two were associated with the viral vector [8] and inactivated vaccination [9]. A fourth type of COVID-19 vaccine is the protein subunit vaccine containing a stable prefusion spike protein of SARS-CoV-2 [10,11]. Herein, we present the first case of DHZ following the protein subunit COVID-19 vaccination and a case of DHZ following mRNA COVID-19 vaccination in elderly, immunocompetent male patients. We have further summarized the characteristics of COVID-19 vaccination-related DHZ in immunocompetent patients through this case report and related literature review.

## 2. Case Reports

**Figure 1:**
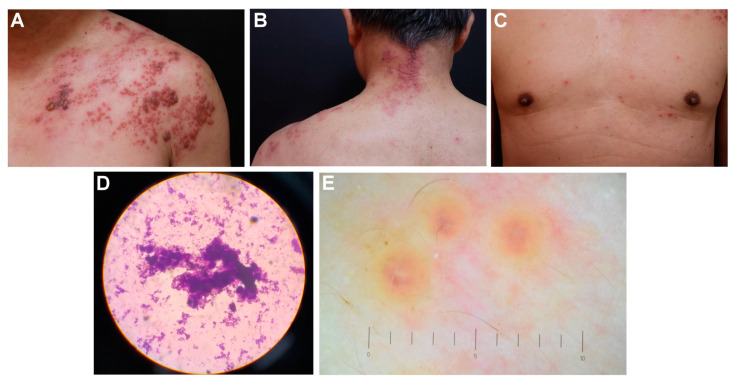
Cutaneous manifestations, Tzanck smear images, and dermoscopic images of case 1. (**A**–**C**) Grouped vesicles over erythematous patches on the left C3–5 dermatomes with multiple disseminated vesicles and erythematous papules on the trunk. (**D**) Tzanck smear revealed multinucleated giant cells. (**E**) Dermoscopic images of the skin lesions revealed central umbilication and an erythematous background.

Case 1 was a 64-year-old immunocompetent male patient. He was generally healthy, without an underlying disease and with no history of immunosuppressant use. He denied any recent contact history or herpes zoster vaccination and could not recall a history of chicken pox. He received three doses of the SARS-CoV-2 spike protein vaccine (MVC-COV1901; Medigen Vaccine Biologics, Taipei, Taiwan). He developed DHZ 1 month after receiving the third dose, and he had painful skin lesions on the left neck, chest, and shoulder. A skin examination revealed grouped vesicles over erythematous patches on the left C3–5 dermatomes (Figure 1A,B) with multiple disseminated vesicles and erythematous papules on the trunk (Figure 1C) as well as extremities. Tzanck smears revealed multinucleated giant cells (Figure 1D). A dermoscopy revealed central umbilication on an erythematous background (Figure 1E). The diagnosis of SARS-CoV-2 spike protein vaccination-related DHZ with left C3–5 predominant was established. He did not have laboratory data to indicate whether VZV infection was previous or current. Oral famciclovir (250 mg, three times/day for 7 days) was administered, and the patient achieved total remission of skin lesions without visceral involvement or severe post-herpetic neuralgia.

**Figure 2:**
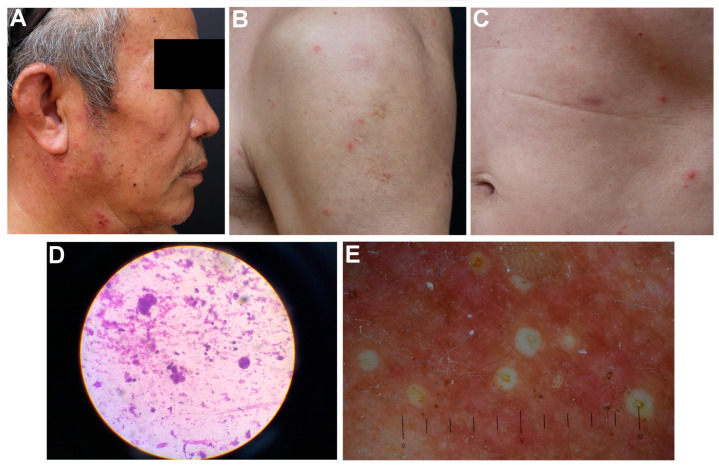
Cutaneous manifestations, Tzanck smear images, and dermoscopic images of case 2. (**A**–**C**) Grouped vesicles over erythematous patches on the right cranial nerve V1–3 dermatomes with multiple disseminated vesicles and erythematous papules on the extremities and trunk. (**D**) Tzanck smear revealed multinucleated giant cells. (**E**) Dermoscopic images of the skin lesions revealed central umbilication and an erythematous background.

Case 2 was a 67-year-old immunocompetent male patient. He was also generally healthy, without a remarkable underlying disease and with no history of immunosuppressant use. He denied any recent contact history or herpes zoster vaccination and could not recall a history of chicken pox. He received two doses of the mRNA-1273 SARS-CoV-2 vaccine (Moderna Inc., Cambridge, MA, USA). He developed DHZ 7 days after receiving the second dose. A skin examination revealed grouped vesicles over the right scalp, periorbital region, nose tip, cheek, and preauricular region, indicating right cranial nerve V1–3 dermatomes (Figure 2A). In addition, >50 vesicles were found on the left cheek, extremities (Figure 2B), and trunk (Figure 2C), which were confirmed using both Tzanck smears with multinucleated giant cells (Figure 2D) and a dermoscopy with central umbilication on an erythematous background (Figure 2E). The diagnosis of mRNA COVID-19 vaccination-related DHZ with right V1–3 predominant was established. Laboratory data indicated positive VZV immunoglobulin G, negative immunoglobulin M, and negative DNA of VZV, suggesting previous but not current infection. Oral famciclovir (250 mg, three times/day for 7 days) was administered, and the patient achieved total remission of skin lesions without visceral involvement or severe post-herpetic neuralgia.

## 3. Discussion

DHZ typically occurs in immunocompromised persons, but it remains uncommon in healthy persons [5,12]. This report describes the first case of DHZ following the protein subunit COVID-19 vaccination and a case of DHZ following mRNA COVID-19 vaccination; both cases were immunocompetent patients. The clinical course and prognosis of these two cases are benign. Both of the patients achieved remission after being treated with systemic anti-viral therapies.

**Figure 3:**
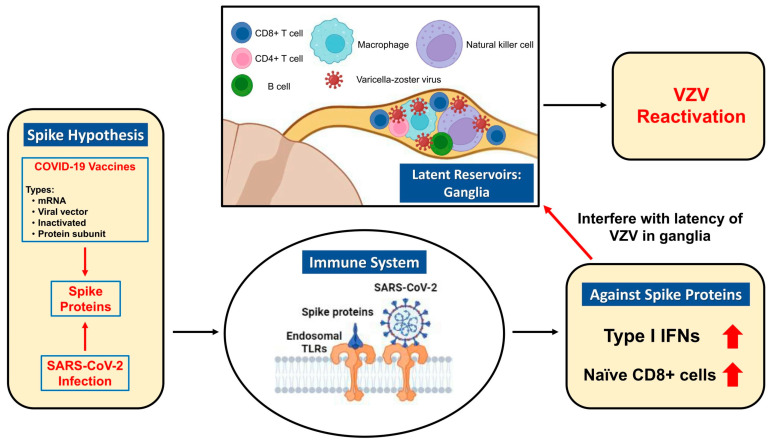
Schematic summary of the proposed mechanisms underlying COVID-19 vaccination- or infection-related VZV reactivation. During VZV latency in human ganglia, most T cells infiltrating ganglia are CD8+, but CD4+ T cells, natural killer cells, B cells, and macrophages can also be detected [14]. Spike proteins of SARS-CoV-2 are the common triggers for VZV reactivation secondary to different types of COVID-19 vaccination or COVID-19 infection [20]. Spike proteins stimulate innate immunity through endosomal Toll-like receptors, leading to the induction of type I IFNs [3,15] and the shift of naïve CD8+ T cells [13]. These immunomodulations interfere with the latency of VZV in ganglia and result in VZV reactivation. TLRs, Toll-like receptors; IFNs, interferons; VZV, varicella-zoster virus.

The pathogenic mechanism underlying COVID-19 vaccination-related DHZ remains unclear. It is generally believed that COVID-19 vaccines produce certain immunomodulations leading to VZV reactivation. For example, some investigators [2,13] postulated that VZV-specific CD8+ cells temporarily lose control of VZV after the shift of naïve CD8+ cells in the setting of a COVID-19 vaccination. CD8+ T cells were found to be prominent in human sensory ganglia following natural VZV reactivation [14]. Other investigators [3,15] proposed that the COVID-19 vaccines can stimulate innate immunity through Toll-like receptors 3 and 7, and the induction of type I interferons and potent inflammatory cytokines, which may negatively affect antigen expression, potentially interfering with the suppression of VZV reactivation. Type I interferon signaling pathways were considered to play important roles in protecting against VZV reactivation [16,17]. Whatever the mechanism, the trigger for VZV reactivation resulting in DHZ should be consistent among different types of COVID-19 vaccinations. Indeed, this and previous studies found that DHZ could occur after the protein subunit, mRNA-based [6,7], viral vector [8], and inactivated vaccinations [9]. In addition, DHZ cases secondary to natural infections with SARS-CoV-2 have been reported [18,19]. These findings indicate that the spike protein of SARS-CoV-2 could be the common trigger for VZV reactivation in these settings. This notion is consistent with the “spike hypothesis” proposed recently to explain why different types of COVID-19 vaccinations produce the same adverse events [20]. Figure 3 shows a schematic diagram illustrating the proposed pathogenic mechanism underlying COVID-19 vaccination- or infection-related VZV reactivation. Apart from VZV reactivation, several case reports documented a proven VZV infection following COVID-19 vaccination [21,22] or infection [23]. Nevertheless, whether VZV reactivation or infection after COVID-19 vaccinations has a causal relationship or is just coincidental is still debated. Further investigations are required to elucidate the relationship between different types of COVID-19 vaccination and VZV reactivation.

With the addition of two cases from this study, only six cases of DHZ following COVID-19 vaccinations have been reported in immunocompetent patients [6,7,8,9]. The major characteristics of these cases are summarized in Table 1. Among the six cases in this review, three cases were secondary to mRNA COVID-19 vaccination probably because this type of vaccine is the most widely used [20]. Notably, all six cases were elderly male individuals as VZV reactivation is known to be more frequent in elderly persons because of their diminished cell-mediated immunity, which is also known as immunosenescence [3]. Moreover, most of the cases had DHZ following the second or third dose of vaccination. The interval between vaccination and the onset of DHZ in patients with protein subunit vaccine is longer than that in patients with other types of vaccine. Whether this long interval is within the wide range of time to the onset of VZV reactivation following COVID-19 vaccination [4] or infection [24], or whether it is unique to the protein subunit vaccination, remains to be investigated. Finally, all six cases in this review are manageable with the treatment with anti-viral therapy, so VZV reactivation should not be a serious concern for patients and physicians in this setting. However, the awareness of the possible reactivation of other fatal viruses, such as hepatitis B or C virus, following COVID-19 vaccines [25,26] is necessary.

## 4. Conclusions

As a rare cutaneous adverse event in immunocompetent patients, DHZ could be secondary not only to mRNA COVID-19 vaccination but also to the protein subunit COVID-19 vaccination. The spike protein of SARS-CoV-2 could be the common trigger for the reactivation of VZV among different types of vaccinations. Thus, prompting diagnosis and anti-viral therapy are necessary to avoid severe and prolonged post-herpetic neuralgia.

## Figures and Tables

**Table 1:** Reported cases of disseminated herpes zoster following COVID-19 vaccination in immunocompetent patients.

First Author	Age/Sex	Type of Vaccine	Brand Name/Company	Dose	Interval (Days)	Treatment	Chronic Disease
Lin (our case)	64/Male	Protein subunit	MVC-COV1901/Medigen	3	30	Oral famciclovir	Unremarkable
Lin (our case)	67/Male	mRNA	mRNA-1273/Moderna	2	7	Oral famciclovir	Unremarkable
Zengarini	65/Male	mRNA	BNT162b2/Pfizer-BioNTech	3	7	Oral acyclovir	Unremarkable
Riazuddin	65/Male	mRNA	BNT162b2/Pfizer-BioNTech	3	4	Intravenous acyclovir	Diabetes
Jiang	79/Male	Viral vector	AZD 1222/AstraZeneca	1	4	Intravenous acyclovir	Parkinsonism
Zhang	65/Male	Inactivated	BBV 152/Covaxin	3	7	Oral valacyclovir	Hypertension

## Data Availability

Data are unavailable due to privacy or ethical restrictions.
